# Fragile streak goals elicit multimodal pressure responses and late-stage performance decline

**DOI:** 10.1016/j.isci.2026.118040

**Published:** 2026-10-27

**Authors:** Kagari Yamada, Kazushi Tsutsui, Kazutoshi Kudo

**Affiliations:** 1 Graduate School of Arts and Sciences, The University of Tokyo, Tokyo, Japan; 2 Center for Information and Neural Networks (CiNet), National Institute of Information and Communications Technology (NICT), Suita, Japan; 3 Research Fellow of Japan Society for the Promotion of Science, Tokyo, Japan; 4 Graduate School of Informatics, Nagoya University, Nagoya, Japan; 5 Center for Advanced Intelligence Project, RIKEN, Tokyo, Japan

**Keywords:** psychological pressure, choking under pressure, heart rate, pupil size, motor control, consecutive successes

## Abstract

Understanding how psychological pressure alters motor performance requires paradigms that reliably induce high-pressure states; yet, laboratory studies often fail to elicit states strong enough to affect performance. We tested whether a fragile goal structure, in which failure resets progress toward consecutive successes, creates a pressure-related state during a task requiring sustained force control. Participants pursued ten consecutive successes or 100 successes irrespective of sequence. Under the streak goal, heart rate, pupil size, and perceived pressure rose as participants approached their maximum streak; under the total goal, heart rate and pupil size showed little modulation. Critically, performance declined at the maximum streak under the streak goal, whereas the total goal showed no corresponding late-stage decline. Movement-vigor measures did not reliably vary with streak progress, suggesting that the decline was not explained by faster, stronger movements. Fragile streak goals provide a scalable context for studying pressure-related responses and goal-structure-dependent, choking-like performance decline in controlled tasks.

## Highlights

• Perceived pressure rises as consecutive successes accumulate under a streak goal
• Heart rate and pupil size rise as streaks lengthen under streak but not total goals
• Streak-goal performance drops at the maximum streak, when pressure and arousal peak
• Movement vigor indices (speed, reaction time) do not vary reliably with streak length

## Graphical abstract

**Figure:**
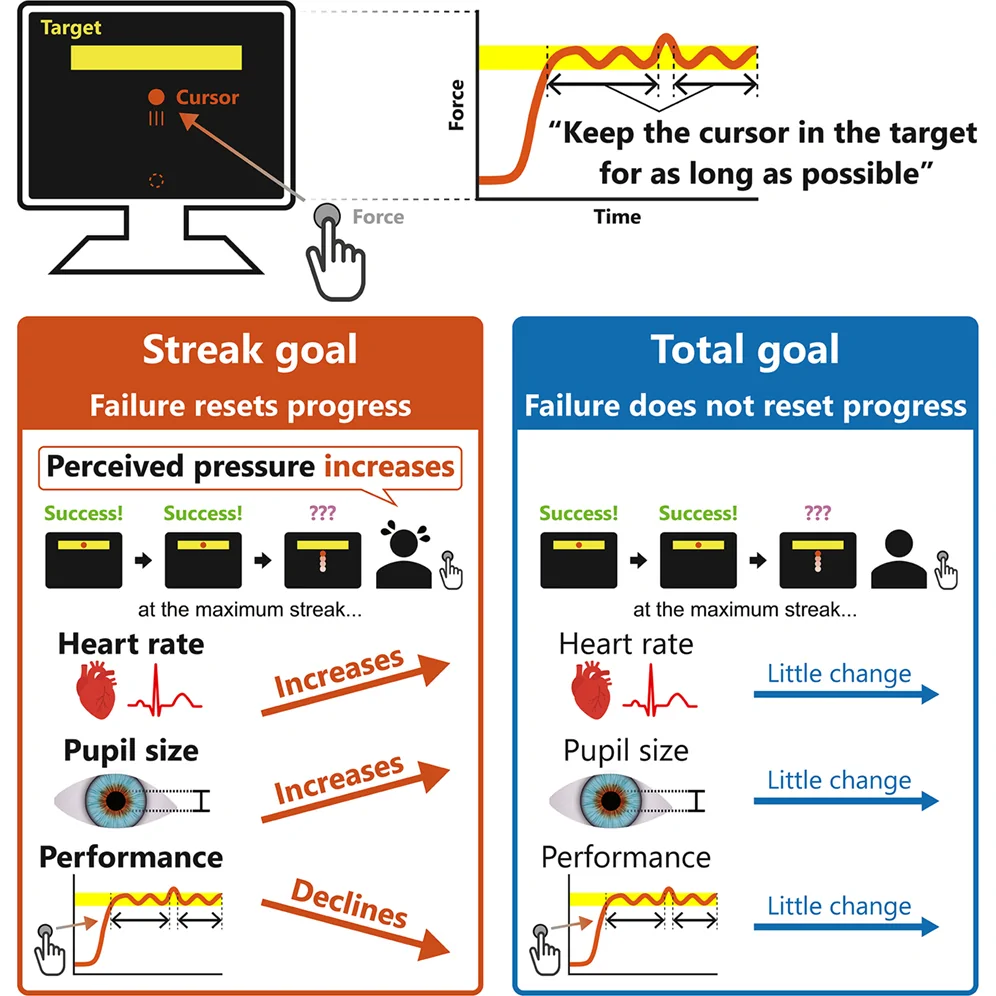

## Teaser

Applied sciences; Behavioral neuroscience

## Introduction

In situations where high financial rewards or social evaluations depend on an individual’s performance, individuals sometimes struggle to perform as intended.^1^ This phenomenon, known as “choking under pressure,” is defined as “performance decrements due to psychological pressure.”^2^

However, observing performance changes under high psychological pressure in laboratory settings is often challenging. In real-world high-stakes situations, such as piano competitions held before large audiences and judges, individuals exhibit substantial physiological and psychological responses.^3^ By contrast, common laboratory manipulations (e.g., monetary incentives or mild social evaluation) rarely elicit responses of comparable magnitude.^4^^,^^5^^,^^6^^,^^7^ Because standard laboratory manipulations rarely push humans into truly high-arousal states, researchers may sample only a limited range of pressure levels.^8^ This methodological limitation contributes to the lack of clarity regarding how motor performance changes under severe psychological pressure.

In our recent study,^9^ we introduced an experimental paradigm in which arousal was progressively induced by setting consecutive success as a goal. When participants aimed to achieve ten consecutive successes, their heart rate, which is a widely used objective measure of arousal often associated with psychological pressure, increased exponentially as the number of successes accumulated. The magnitude of this heart rate increase was substantially greater than that reported in previous studies using more traditional pressure manipulations. However, because heart rate reflects physiological arousal rather than psychological states per se, that study alone could not fully disentangle whether the observed increase in arousal specifically reflected heightened psychological pressure. Inferring a specific psychological state from physiological data alone requires caution, given the issue of reverse inference,^10^^,^^11^ as other states besides pressure can also influence heart rate.^12^

To address this limitation and rigorously validate the paradigm, the present study incorporated two additional measures. First, we measured pupil size, which reflects emotional arousal linked to sympathetic nervous system activity^13^ and closely tracks trial-by-trial changes in brain state,^14^ making it a suitable index for fluctuating arousal under pressure.^15^^,^^16^ Second, and more critically, we directly assessed participants’ perceived pressure through subjective ratings. This multimodal approach allows us to evaluate the pressure-related state elicited by the goal-structure manipulation without relying on a single physiological index.

Using this goal-structure-based paradigm, we aimed to test whether a fragile streak goal can elicit convergent subjective and physiological pressure-related responses, and whether this high-pressure fragile-goal context is accompanied by changes in motor performance. Rather than treating psychological pressure as isolated from task structure, we conceptualized the streak goal as a fragile goal context in which perceived pressure and physiological arousal may co-occur. We therefore focused on two questions: whether the streak-goal structure produces pressure-related responses, and whether performance deteriorates specifically at the late stage of the streak-goal context.

## Results

Participants were instructed to maintain the cursor within the target area for as long as possible by applying force with their index fingers. This required moving the cursor into the target area as quickly as possible and maintaining its position within the target (Figure 1A). To induce pressure-related responses, we employed the consecutive success paradigm.^9^ Participants were randomly assigned to one of two groups: the consecutive group or the total group. The consecutive group (*n* = 22) was instructed to achieve 10 consecutive successes, whereas the total group (*n* = 22) was instructed to achieve a total of 100 successes, regardless of sequence (Figures 1B and 1C). In the consecutive group, the counter displayed at trial onset reset to zero after any failure, whereas in the total group the displayed count tracked cumulative successes and therefore remained unchanged after failures. This design allowed us to examine whether a fragile streak-goal structure was accompanied by pressure-related subjective and physiological responses and performance changes beyond those observed when successes accumulated without reset.

**Figure 1:**
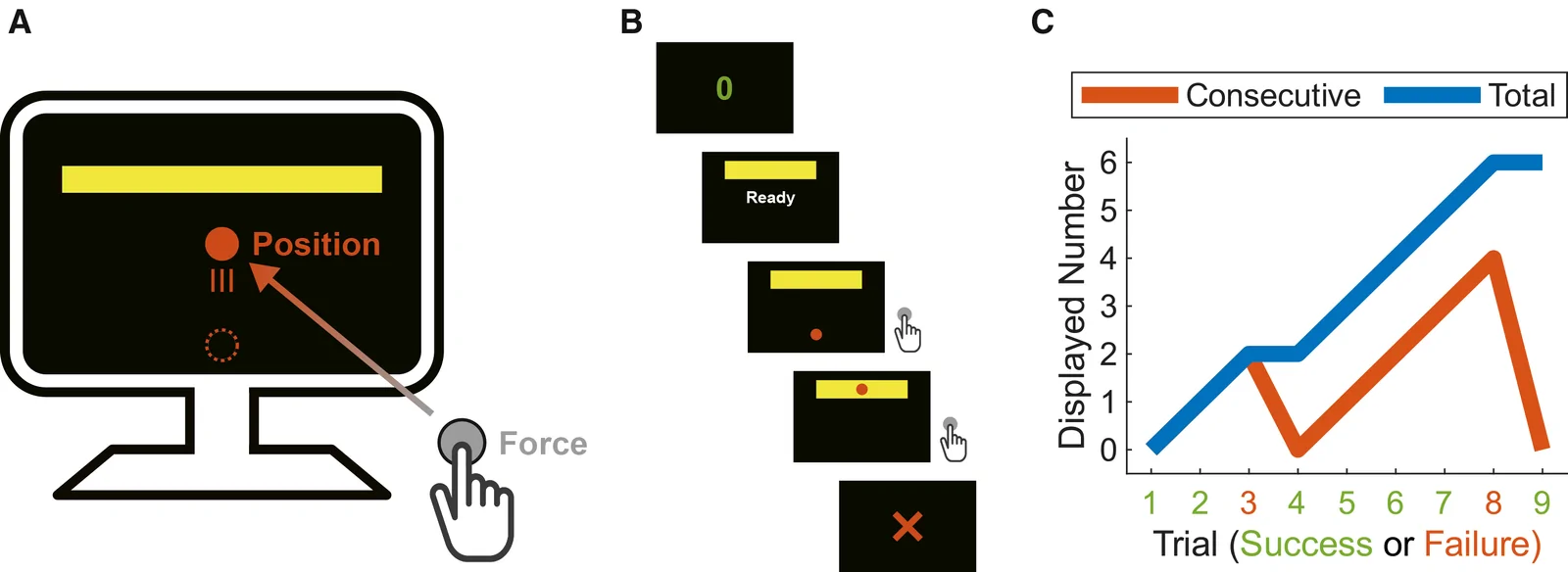
Task and experimental design (A) Participants performed a task requiring delicate force production. A cursor displayed on the screen moved vertically according to the magnitude of the participant’s exerted force. (B) Sequence of procedures in each trial. First, the participant’s current number of consecutive successes in the consecutive group or total successes in the total group was displayed. Then, after a variable interval, participants were required to exert force to move the cursor to the target as quickly as possible and maintain it within the target. Finally, visual feedback of success or failure, determined by the duration the cursor remained within the target, was provided to the participant. (C) Illustration of the number displayed at the beginning of each trial, assuming a hypothetical sequence of successful and unsuccessful trials as shown in the figure. In the consecutive group, the displayed count reflected the current streak and reset to zero after a failure, whereas in the total group it reflected cumulative successes and therefore did not reset after failures. Even with the same sequence of outcomes, the numbers displayed at the start of each trial differed between the groups.

### Pressure-related responses increased with streak proximity under the streak goal

The occurrence of a psychological pressure state was assessed using physiological measures of arousal that are sensitive to pressure, namely heart rate^4^^,^^17^^,^^18^^,^^19^ and pupil size,^15^^,^^16^ together with subjective ratings of perceived pressure. To link these measures to progress within a streak in a way that is comparable across participants, we first computed trial-wise averages for each number of consecutive successes and then grouped trials into three within-subject streak-proximity conditions defined relative to each participant’s maximum number of consecutive successes (see STAR Methods for details). In brief, trials were classified as far, near, or max depending on whether they occurred far from, just before, or at the participant’s maximum streak.

Physiological indices were consistent with increased pressure-related arousal when participants pursued a consecutive-success goal. Heart rate showed a significant main effect of streak proximity (*F* [1.31, 52.34] = 16.46, *p* < 0.001, *η*_*G*_^*2*^ = 0.047) and a group × streak proximity interaction (*F* [1.31, 52.34] = 17.44, *p* < 0.001, *η*_*G*_^*2*^ = 0.049) (Figure 2A). In the consecutive group, heart rate increased monotonically from the far to near and max conditions (*F* [1.23, 24.63] = 19.31, *p* < 0.001, *η*_*G*_^*2*^ = 0.137), with all pairwise comparisons significant (far vs. near: *F* [1, 20] = 16.51, *p* < 0.001, *η*_*G*_^*2*^ = 0.095; near vs. max: *F* [1, 21] = 16.89, *p* < 0.001, *η*_*G*_^*2*^ = 0.033). In contrast, heart rate did not vary reliably with streak proximity in the total group (*F* [1.58, 31.50] = 0.28, *p* = 0.70, *η*_*G*_^*2*^ = 0.001).

**Figure 2:**
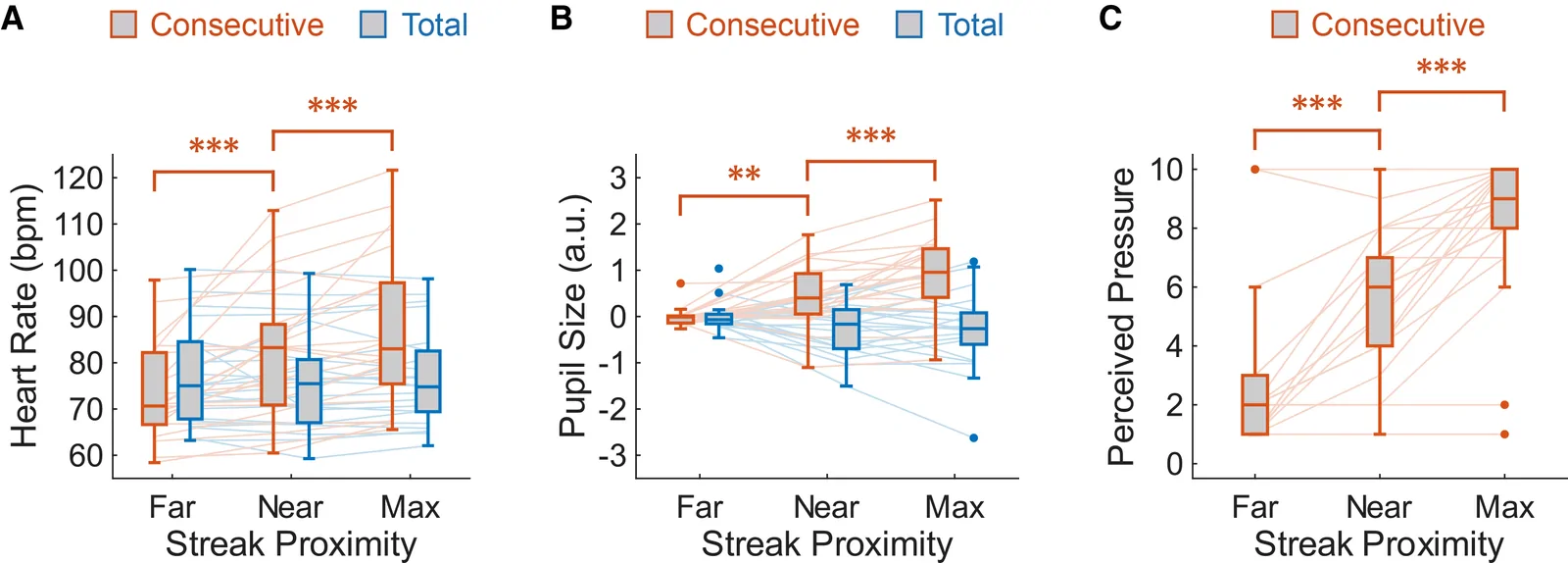
Streak proximity was accompanied by increased pressure-related responses under the streak goal (A) Heart rate across the three streak-proximity conditions (far, near, max) in the consecutive and total groups. (B) Pupil size across the three streak-proximity conditions (far, near, max) in the two groups. (C) Retrospective ratings of perceived pressure across the three streak-proximity conditions (far, near, max) in the consecutive group. Streak proximity (far, near, max) was defined relative to each participant’s maximum number of consecutive successes (see STAR Methods). In (A and B), red denotes the consecutive group and blue denotes the total group (C includes the consecutive group only). Box-and-whisker plots show the median (center line) and interquartile range (box); whiskers extend to 1.5× the interquartile range and points denote outliers. Thin lines connect individual participants across streak-proximity conditions. Asterisks indicate significant planned pairwise comparisons (∗∗*p* < 0.01, ∗∗∗*p* < 0.001).

A similar pattern was observed for pupil size. Overall pupil size exhibited significant main effects of group (*F* [1, 38] = 18.23, *p* < 0.001, *η*_*G*_^*2*^ = 0.225) and streak proximity (*F* [1.46, 55.38] = 5.50, *p* < 0.05, *η*_*G*_^*2*^ = 0.054), as well as a group × streak proximity interaction (*F* [1.46, 55.38] = 15.09, *p* < 0.001, *η*_*G*_^*2*^ = 0.135) (Figure 2B). In the consecutive group, pupil size increased substantially across streak proximity levels (*F* [1.47, 27.93] = 19.32, *p* < 0.001, *η*_*G*_^*2*^ = 0.278), with all pairwise comparisons significant (far vs. near: *F* [1, 19] = 11.14, *p* < 0.01, *η*_*G*_^*2*^ = 0.203; near vs. max: *F* [1, 20] = 18.86, *p* < 0.001, *η*_*G*_^*2*^ = 0.103). In the total group, pupil size showed no reliable modulation by streak proximity (*F* [1.41, 26.75] = 1.40, *p* = 0.26, *η*_*G*_^*2*^ = 0.029).

To confirm that these physiological effects were not artifacts of the far/near/max binning procedure, we also fitted trial-level mixed-effects models using the raw number of consecutive successes as a continuous predictor. These analyses yielded the same qualitative conclusion (Figures S1A and S1B).

Since psychological states other than perceived pressure can also influence physiological measures, it is not possible to determine participants’ psychological state solely based on changes in heart rate and pupil size.^12^ Previous studies have employed methods that validate pressure manipulation using both subjective and objective measures.^4^^,^^17^^,^^18^^,^^19^ Therefore, we assessed participants’ perceived pressure using a questionnaire administered after the main session in the consecutive group and confirmed through subjective measures that their perceived pressure increased with streak proximity (*F* [1.44, 30.31] = 51.7, *p* < 0.001, *η*_*G*_^*2*^ = 0.440), with all pairwise comparisons significant (far vs. near: *F* [1, 21] = 37.66, *p* < 0.001, *η*_*G*_^*2*^ = 0.252; near vs. max: *F* [1, 21] = 36.89, *p* < 0.001, *η*_*G*_^*2*^ = 0.213) (Figure 2C). The convergence of self-reported and physiological measures indicated that the experimental manipulation in this study successfully induced a psychological pressure state.

### Performance declined at the maximum streak only under the fragile streak goal

We next examined whether the fragile streak-goal context, which elicited strong subjective and physiological pressure-related responses, was accompanied by changes in task performance. Because the consecutive and total groups differed in goal structure as well as in pressure-related responses, we did not assume that all performance changes across streak proximity were uniquely pressure-driven. Instead, we focused on whether performance changes common to both groups could be distinguished from a late-stage change specific to the fragile streak-goal context. The duration for which the cursor remained within the target area was recorded as a score (Figure 3A). Based on this score, each trial was classified as either a success or a failure. Participants were informed of this scoring criterion and were instructed to maximize their scores. Accordingly, the score can be regarded as a measure of performance in the present experiment.

**Figure 3:**
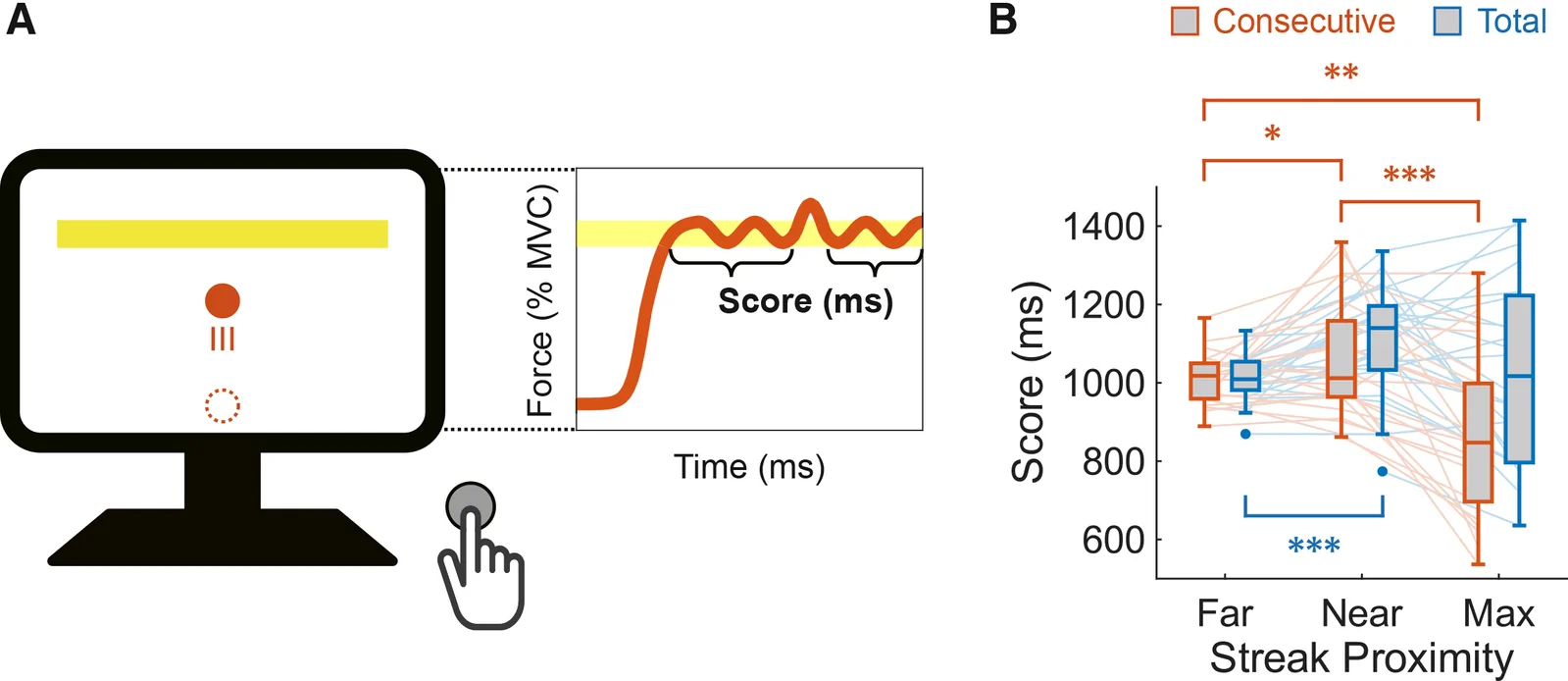
Streak proximity and performance (A) Schematic of the performance measure. The score was defined as the time (ms) that the cursor remained fully within the target during each trial; trials with score ≥1,000 ms were counted as successes. (B) Performance scores across the three streak-proximity conditions (far, near, max) in the consecutive (red) and total (blue) groups. Streak proximity was defined relative to each participant’s maximum number of consecutive successes (see STAR Methods). Red denotes the consecutive group and blue denotes the total group. Box-and-whisker plots show the median (center line) and interquartile range (box); whiskers extend to 1.5× the interquartile range and points denote outliers. Thin lines connect individual participants across streak-proximity conditions. Asterisks indicate significant planned pairwise comparisons (∗*p* < 0.05, ∗∗*p* < 0.01, ∗∗∗*p* < 0.001).

A 2 (group: consecutive vs. total) × 3 (streak proximity: far, near, max) mixed ANOVA on the score revealed significant main effects of group (*F* [1, 40] = 4.82, *p* < 0.05, *η*_*G*_^*2*^ = 0.062) and streak proximity (*F* [1.51, 60.46] = 13.25, *p* < 0.001, *η*_*G*_^*2*^ = 0.129), as well as a group × streak proximity interaction (*F* [1.51, 60.46] = 4.62, *p* < 0.05, *η*_*G*_^*2*^ = 0.049). Performance varied with streak proximity levels in both groups but with distinct patterns (Figure 3B).

In the consecutive group, performance changed markedly across streak proximity levels (*F* [1.64, 32.89] = 12.31, *p* < 0.001, *η*_*G*_^*2*^ = 0.222). Scores increased from far to near streak proximity (*F* [1, 20] = 4.45, *p* < 0.05, *η*_*G*_^*2*^ = 0.072) and then declined at max streak proximity (*F* [1, 21] = 18.77, *p* < 0.001, *η*_*G*_^*2*^ = 0.223). Notably, scores at max streak proximity were significantly different from those at far (*F* [1, 20] = 10.12, *p* < 0.01, *η*_*G*_^*2*^ = 0.154). In the total group, performance also differed across streak proximity levels (*F* [1.36, 27.22] = 5.18, *p* < 0.05, *η*_*G*_^*2*^ = 0.101), driven by improved scores from far to near streak proximity (*F* [1, 20] = 28.06, *p* < 0.001, *η*_*G*_^*2*^ = 0.276). However, unlike in the consecutive group, there was no reliable decline in performance from near to max streak proximity (*F* [1, 21] = 3.86, *p* = 0.09, *η*_*G*_^*2*^ = 0.050), and performance at max did not differ from far (*F* [1, 20] = 0.68, *p* = 0.42, *η*_*G*_^*2*^ = 0.011). Thus, the far-to-near improvement was shared across the two groups and may reflect task-common factors such as recent successful performance, adaptation, or increasing familiarity within a streak. In contrast, the near-to-max decline was observed only when the streak itself determined task termination. Trial-level mixed-effects analyses provided converging evidence for this late-stage downturn and indicated that it was not solely attributable to the binning procedure (Figure S1C).

Together, the performance decline at max occurred in the same condition in which perceived pressure, heart rate, and pupil size were highest. We interpret the late-stage drop as a performance decline associated with a high-pressure, fragile streak-goal context.

### No evidence for increased movement vigor under heightened pressure

It has been well established that human motor behavior changes when larger rewards are at stake—that is, when motivation is heightened—and these changes have been attributed to increases in movement vigor.^20^ Increased vigor is associated with reduced reaction times,^21^^,^^22^ faster movement speeds,^23^^,^^24^^,^^25^ and greater force production.^26^^,^^27^ In the present study, we investigated whether the observed behavioral changes could similarly be explained by a change in movement vigor as streak proximity increased.

To this end, we examined whether streak proximity was associated with changes in reaction time, peak movement speed, or force magnitude. For each dependent measure, we averaged trials within the far, near, and max streak-proximity conditions for each participant and submitted these values to a 2 (group: consecutive, total) × 3 (streak proximity: far, near, max) mixed ANOVA.

There was no evidence that reaction time shortened with streak proximity. For reaction time (Figure 4A), the mixed ANOVA revealed no significant main effects of group (*F* [1, 39] = 1.59, *p* = 0.22, *η*_*G*_^*2*^ = 0.034) or streak proximity (*F* [1.29, 50.43] = 0.29, *p* = 0.65, *η*_*G*_^*2*^ = 0.001), and no group × streak proximity interaction (*F* [1.29, 50.43] = 0.40, *p* = 0.58, *η*_*G*_^*2*^ = 0.001). Consistent with this, one-way repeated-measures ANOVAs within each group showed no reliable variation in reaction time across streak-proximity levels in either the consecutive group (*F* [1.21, 22.95] = 0.54, *p* = 0.50, *η*_*G*_^*2*^ = 0.004) or the total group (*F* [1.36, 27.25] = 0.02, *p* = 0.95, *η*_*G*_^*2*^ = 0.000).

**Figure 4:**
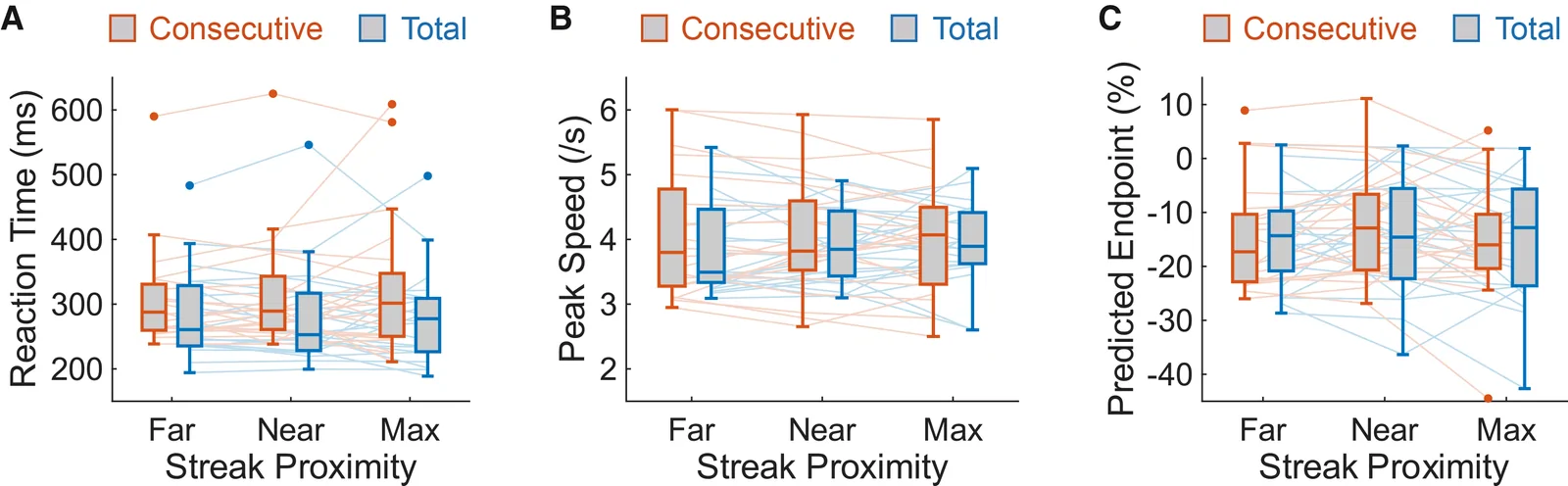
No evidence for increased movement vigor as a function of streak proximity (A) Reaction time across streak-proximity conditions in the consecutive (red) and total (blue) groups. (B) Peak speed across streak-proximity conditions in the two groups. (C) Force-related output index (predicted endpoint relative to the target) across streak-proximity conditions in the two groups. Streak proximity was defined relative to each participant’s maximum number of consecutive successes (see STAR Methods). In (A–C), red denotes the consecutive group and blue denotes the total group. Box-and-whisker plots show the median (center line) and interquartile range (box); whiskers extend to 1.5× the interquartile range and points denote outliers. Thin lines connect individual participants across streak-proximity conditions.

Similarly, streak proximity was not associated with increased movement speed. For peak movement speed (Figure 4B), the mixed ANOVA again yielded no significant main effects of group (*F* [1, 39] = 0.41, *p* = 0.53, *η*_*G*_^*2*^ = 0.009) or streak proximity (*F* [1.77, 69.10] = 0.02, *p* = 0.97, *η*_*G*_^*2*^ = 0.000), and no group × streak proximity interaction (*F* [1.77, 69.10] = 0.54, *p* = 0.56, *η*_*G*_^*2*^ = 0.001). Within-group analyses confirmed the absence of reliable effects of streak proximity on peak speed in both the consecutive group (*F* [1.39, 26.34] = 0.27, *p* = 0.69, *η*_*G*_^*2*^ = 0.001) and the total group (*F* [1.92, 38.50] = 0.30, *p* = 0.73, *η*_*G*_^*2*^ = 0.003).

Finally, we examined force-related output, indexed by the predicted endpoint derived from acceleration-phase kinematics (see STAR Methods), relative to the target (Figure 4C). The mixed ANOVA showed no significant main effects of group (*F* [1, 39] = 0.04, *p* = 0.84, *η*_*G*_^*2*^ = 0.001) or streak proximity (*F* [1.83, 71.55] = 1.25, *p* = 0.29, *η*_*G*_^*2*^ = 0.007) and no interaction (*F* [1.83, 71.55] = 0.10, *p* = 0.89, *η*_*G*_^*2*^ = 0.001). One-way ANOVAs within each group likewise showed no reliable modulation of predicted endpoint by streak proximity in either the consecutive group (*F* [1.20, 22.80] = 1.03, *p* = 0.33, *η*_*G*_^*2*^ = 0.012) or the total group (*F* [1.87, 37.36] = 0.33, *p* = 0.71, *η*_*G*_^*2*^ = 0.004).

Together, these results indicate that the motor changes typically associated with increased movement vigor—shorter reaction times, faster movements, and greater force output—were not elicited as participants approached their maximum streak. Instead, the performance changes associated with increasing streak proximity under the fragile streak-goal context appear to arise from other facets of motor control.

To probe candidate motor-control mechanisms beyond movement vigor, we conducted exploratory kinematic analyses motivated by distinctions between predictive and sensory-driven control (Figure S2). In addition, we examined whether individual differences in physiological and subjective pressure-related responses covaried and predicted the within-streak performance changes (Figure S3). These analyses yielded largely null findings: feedforward/feedback-related kinematic indices did not show robust streak-proximity-dependent signatures at the group level, and individual differences in pressure indices did not reliably predict performance changes.

## Discussion

Observing and understanding performance decrements under psychological pressure may have been hindered by the methodological difficulty of inducing truly high-pressure states in laboratory settings. By imposing a “fragile” goal structure—requiring a streak of ten consecutive successes—we elicited convergent subjective and physiological pressure-related responses and observed a late-stage performance decline.

A central contribution of this work is a more rigorous validation of the consecutive-success paradigm as a goal-structure-based method for eliciting a pressure-related task state. In prior work,^9^ pressure was inferred primarily from heart rate, leaving room for alternative interpretations because physiological indices alone do not uniquely specify psychological states.^10^^,^^11^^,^^12^ Here, we triangulated pressure using heart rate, pupil size, and perceived pressure ratings—measures that are widely used as indices of pressure, competitive anxiety, and arousal in performance settings.^4^^,^^15^^,^^16^^,^^17^^,^^18^^,^^19^ All three rose systematically with increasing streak proximity when success had to be maintained as a streak, whereas physiological indices showed little systematic modulation with increasing streak proximity when the goal was to accumulate a fixed number of successes irrespective of sequence. This comparison indicates that it is not repeated success per se, but the coupling between each attempt and the preservation of a streak, that robustly amplifies pressure. Perceived pressure was not assessed in the total group, precluding a direct subjective comparison between goal structures. Nevertheless, the absence of comparable heart rate and pupil size modulation in that group does not support a similarly increasing multimodal pressure-related state.

The observed performance trajectory under the streak-based goal provides critical insights into how motor control changes in high-stakes environments. Within the streak-based goal structure, as participants approached the end of a required streak (higher streak proximity), performance improved from low to intermediate streak proximity. However, because an equivalent improvement was observed in the total group, this initial rise may reflect task learning or general adaptation.^28^^,^^29^^,^^30^^,^^31^ Critically, however, performance declined at high streak proximity—where our physiological and subjective measures indicate that pressure was highest—when success depended on preserving the streak, whereas performance under the total-success goal tended to plateau rather than collapse. This dissociation aligns with the idea that choking is most likely when failures are especially costly or salient—mirroring real-world “one-mistake” contexts such as decisive shots and match points—rather than whenever incentives or repetition are present.^32^^,^^33^^,^^34^^,^^35^^,^^36^ At the same time, the streak goal necessarily increased the cost and salience of failure^32^^,^^33^ and may have altered attentional,^37^ motivational,^38^ and strategic demands.^39^ These factors are intertwined with the pressure-related task state induced by the paradigm, and the present design cannot isolate their individual contributions to the late-stage decline. We therefore interpret the decline as occurring within a high-pressure, fragile streak-goal context rather than as an effect caused by psychological pressure alone.

We next asked whether the performance decline under high pressure could be explained by changes in movement vigor. In many reward and motivation contexts, higher stakes invigorate actions, yielding reduced reaction times,^21^^,^^22^ faster movement speeds,^23^^,^^24^^,^^25^ and greater force production.^26^^,^^27^ Our data provided no evidence for this account: reaction time, peak cursor speed, and force-related indices did not vary reliably with streak proximity. These null effects argue against a simple explanation in which high pressure makes participants “move faster/stronger and therefore fail,” and instead point to other mechanisms. For instance, prior studies suggest that changes in attention and anxiety can alter visuomotor execution without overt invigoration.^17^^,^^40^^,^^41^

To probe candidate motor-control mechanisms more directly, we conducted exploratory kinematic analyses (see supplemental information) motivated by distinctions between predictive and sensory-driven control.^42^^,^^43^ However, the accuracy of feedforward and feedback control did not show consistent modulation by streak proximity at the group level. This stands in contrast to evidence from non-human primates, where high-stakes contexts can yield comparatively systematic behavioral and neural changes.^44^^,^^45^ One possibility is that, in humans, choking reflects heterogeneous “routes” to failure that average out when analyzed at the group level: some individuals may become distractible, others may over-monitor their movements, and still others may change how they weight sensory evidence versus predictions.^2^^,^^37^^,^^46^^,^^47^^,^^48^^,^^49^^,^^50^ However, without direct measures of these internal processes, the present data cannot support any specific mechanism underlying the performance collapse. Consistent with this multifaceted view, correlational analyses of change scores yielded no robust associations among heart rate, pupil size, perceived pressure, and performance, suggesting that pressure-related responses do not necessarily covary across people and may only partially overlap across physiological, subjective, and behavioral channels.^51^^,^^52^^,^^53^ Taken together, these findings suggest that pressure-induced performance changes in humans may not be reducible to a single kinematic mechanism, but can emerge through multiple, partially dissociable routes that vary across individuals—and no consistent pressure-related effects were found in kinematics at the group level.

Interestingly, the performance decline observed here contrasts with our previous findings using a similar consecutive-success paradigm.^9^ In that study, performance improved monotonically as the streak lengthened, even though the goal structure elicited an exponential increase in heart rate comparable to the profound arousal observed in the present study. This discrepancy—where the exact same pressure-inducing framework yields either a linear improvement or a late-stage collapse—suggests that the relationship between pressure and performance is highly task-dependent. Several theoretical and empirical accounts propose that task difficulty dictates whether this relationship is linear or follows an inverted-U curve.^54^^,^^55^^,^^56^ However, the average success rate in the previous study^9^ (51.1 ± 10.1%) and that of the consecutive group in the present study (47.4 ± 10.8%) did not differ substantially. Thus, it is likely that factors other than overall task difficulty determine how pressure shapes performance trajectories. One potential factor is the nature of the motor execution: whereas the previous study^9^ involved discrete force production without continuous visual feedback, the present task required sustained force control guided by continuous visual feedback. While such structural differences in the task may contribute to the divergent outcomes, this remains speculative. Because our analyses could not definitively identify the specific mechanisms underlying the pressure-induced performance changes, discovering the critical variables that determine when pressure gives rise to performance deterioration remains an important avenue for future research.

In sum, requiring a streak of consecutive successes provides a strong and scalable way to induce multimodal pressure in the laboratory, extending earlier demonstrations based on heart rate alone^9^ and generalizing the manipulation to a visuomotor task. This fragile goal context was accompanied by a late-stage performance decline, but neither movement vigor measures nor the present kinematic indices revealed consistent streak-proximity-dependent signatures, and individual differences did not align across physiological, subjective, and behavioral measures. Ultimately, by providing a method to easily induce substantial psychological pressure accompanied by subjective and physiological changes in a controlled environment, this study offers a valuable platform for elucidating the effects of pressure on motor control and its underlying mechanisms.

### Limitations of the study

One conceptual limitation is that the present design cannot dissociate physiological arousal from subjective psychological pressure. Heart rate and pupil size indexed arousal-related responses, whereas perceived-pressure ratings indexed the subjective component of the pressure-related state, but these measures covaried as participants approached their maximum streak. Thus, the data cannot determine whether the late-stage decline was associated primarily with physiological arousal, subjective pressure, or their interaction.

The present study does not yet specify the causal computational or neural mechanisms by which pressure alters motor control. Future work should combine this paradigm with more targeted perturbations that directly test how pressure alters sensory-predictive weighting and control policies—for example, by manipulating visual feedback and error signals,^29^^,^^57^ and by integrating neural measures that have been implicated in choking under pressure.^15^^,^^35^^,^^45^

Finally, the all-male sample limits generalizability. Competitive-anxiety profiles may vary by gender and age, as shown using the Sport Anxiety Scale-2,^58^^,^^59^ and trait anxiety can shape state anxiety and performance under pressure.^60^^,^^61^ Because trait anxiety was not assessed, we could not test whether it contributed to individual differences in the late-stage decline. Future studies should recruit more diverse samples and incorporate validated trait-anxiety measures to test whether these factors moderate subjective, physiological, and behavioral responses under fragile streak-goal contexts.

## Resource availability

### Lead contact

Requests for further information and resources should be directed to and will be fulfilled by the lead contact, Kagari Yamada (kagari-marines@g.ecc.u-tokyo.ac.jp).

### Materials availability

This study did not generate new unique reagents.

### Data and code availability

• The behavioral, physiological, and perceived-pressure data reported in this study have been deposited in the Open Science Framework (OSF) and are publicly available at https://osf.io/fwhe2/.
• The original MATLAB analysis code for this study has been deposited in OSF and is publicly available at https://osf.io/fwhe2/.
• Any additional information required to reanalyze the data reported in this study is available from the lead contact upon request.

## Acknowledgments

We thank the members of the Kudo Laboratory for their helpful discussions and comments, as well as the administrative staff for their support. This work was supported by 10.13039/501100001691KAKENHI (JP22K17673, JP24K02825, JP24H01434, JP25H01237, and JP25KJ1145) from the 10.13039/501100001691Japan Society for the Promotion of Science (10.13039/501100001691JSPS), Strategic Basic Research Programs ACT-X (JPMJAX24LA) from the 10.13039/501100002241Japan Science and Technology Agency, and Kubota endowed fund from Kubota Corporation. K.Y. was supported by 10.13039/501100001691JSPS as a JSPS Research Fellow.

## Author contributions

Conceptualization and methodology, K.Y. and K.K.; investigation, formal analysis, data curation, visualization, and writing – original draft, K.Y.; writing – review and editing, K.T. and K.K.; funding acquisition, K.Y., K.T., and K.K.; resources, K.K.; supervision, K.K. All authors read and approved the final manuscript.

## Declaration of interests

The authors declare no competing interests.

## Declaration of generative AI and AI-assisted technologies in the manuscript preparation process

During the preparation of this work the authors used ChatGPT in order to improve the readability and language of the manuscript. After using this tool/service, the authors reviewed and edited the content as needed and take full responsibility for the content of the published article.

## STAR★Methods

### Key resources table

**Table:** 

REAGENT or RESOURCE	SOURCE	IDENTIFIER
**Deposited data**		

Behavioral, physiological, and perceived-pressure data	OSF	https://osf.io/fwhe2/
Codes	OSF	https://osf.io/fwhe2/

**Software and algorithms**		

MATLAB 2025a	The Math Works, Inc.	https://www.mathworks.com
EMGworks Acquisition system	Delsys	https://delsys.com/
Eyelink 1000 plus	SR Research	https://www.sr-research.com/

### Experimental model and study participant details

44 men (mean age ±SD = 25.7 ± 10.0 years, age range: 18–60 years) participated in this study. According to self-reports, four participants were left-handed. All participants had normal or corrected-to-normal vision. None reported a history of cardiac disease. This study was approved by the Ethical Review Committee for Experimental Research Involving Human Subjects, Graduate School of Arts and Sciences, University of Tokyo. The target sample size (*n* = 44) was determined *a priori* based on our previous work using the consecutive-success paradigm,^9^ which showed robust, progressive increases in heart rate as consecutive successes accumulated, and to allow balanced assignment to the Consecutive and Total groups (*n* = 22 each). Because the sample consisted entirely of men, differences in the study outcomes across sex or gender groups could not be evaluated. All participants provided written informed consent before participating in the study.

### Method details

#### Apparatus

All visual stimuli were controlled using a computer (Dell, XPS) and displayed on a monitor (I-O DATA, KH2500V-ZX2,1920 × 1080 pixels, refresh rate: 240 Hz). The participants sat in front of a desk with a monitor and were instructed to place the elbow of their dominant hand on an armrest, with their wrist on a box, and their index finger on a pressure sensor (TEAC, TU-QR(T)500N-G). Their non-dominant hand was instructed to rest in a relaxed manner, with their legs on the same side.

The force exerted by the participants was converted into a voltage signal by the pressure sensor. The voltage signal was transmitted to a data acquisition device (National Instruments, USB-6218 (BNC)) via a digital indicator (TEAC, TD-700T) and digitally recorded at a sampling frequency of 1000 Hz on a computer running the MATLAB Data Acquisition Toolbox.

Heart rates were measured using an electrocardiographic sensor (Delsys, Delsys Trigno EKG Biofeedback Sensor). Two pre-lubricated disposable ECG electrodes (Nihon Kohden, Dispo Electrode M Vitrode) were attached to each participant. The measured data were digitally recorded at a sampling frequency of 519 Hz using an EMGworks Acquisition system (Delsys). A trigger (Delsys, Trigger Module SP-U02) was used to control the ECG sensor using a computer.

Pupil size was measured with an Eyelink 1000 plus (SR Research) controlled by a computer (Dell, OptiPlex) and recorded at 500 Hz. To improve the quality of the pupil size data, head movement was minimized using a chinrest, and calibration was performed at the start of each block.

#### Procedure

Participants were randomly assigned to one of two groups, Consecutive or Total. The Consecutive group (22 participants, mean age ±SD = 25.1 ± 8.23 years, age range: 19–53 years) was told that the experiment would end after 10 consecutive successes, whereas the Total group (22 participants, mean age ±SD = 26.3 ± 11.4 years, age range: 18–60 years) was told that the experiment would end after 100 successes in total.

After the maximum force exerted on their index fingers was measured, the participants performed a training session (2 blocks). In the training session, success resulted in a 1% of the height of the monitor decrease in the target size for the subsequent trial, while failure led to a 2% of the height of the monitor increase in the target size. The first block started with the target size of 20% of the height of the monitor. The second block started with the target size at the end of the first block. The target size in the main session was calculated by averaging all reversals in the second block in the training session.

After the training session, the main session began. A block consisted of 50 trials. If the 50th trial was successful, the trials in that block continued until failure. Participants took a 5-min break between blocks. The experiment continued until participants achieved 10 consecutive successes in the Consecutive group and until they reached a total of 100 successful trials in the Total group. However, if they could not, the experiment was terminated 90 min after the start of the experiment. The total number of trials for each participant varied depending on their performance and which of the two stopping criteria was met first. The resulting trial counts for each participant are detailed in Tables S1 and S2. Participants were informed of these conditions beforehand.

After the main session, we administered a questionnaire to participants in the Consecutive group in which they retrospectively rated perceived pressure. Retrospective assessment was adopted to avoid interrupting the task and altering participants’ attentional focus or evaluative context during the experiment. Participants rated perceived pressure using a VAS ranging from 1 (“felt no pressure”) to 10 (“felt a lot of pressure”). Participants in the Total group were not aware of the number of consecutive successes, so it would be difficult to rate perceived pressure as a function of streak proximity. Therefore, perceived pressure was not investigated in the Total group.

#### Task

First, the current number of consecutive successes was presented in the Consecutive group, and the total number of successes at that point was presented in the Total group. After that, the word “Ready” appeared at the center of the monitor, and a target was displayed. The lower edge of the target was positioned 70% of the height of the monitor from the bottom of the monitor and the target size was based on performance during training session. During this phase, participants were instructed not to exert any force on the pressure sensor and the sensors were calibrated. A cursor would be positioned at the center of the target when 10% of their maximum force was exerted. Then “Ready” disappeared, and the cursor appeared 10% of the height of the monitor from the bottom of the monitor. Participants were instructed to move the cursor representing the force exerted by them into the target as quickly as possible upon the cursor appearance, and to hold the cursor within the target until it disappeared. Real-time feedback of the force exerted by participants was provided as the cursor moving on the monitor. The cursor disappeared 2000 ms after its appearance. A trial was classified as successful if the cursor remained within the target for at least 1000 ms within this interval. Finally, participants received visual feedback on whether they could keep their cursor within the target for more than 1000 ms. All visual stimuli and programs were created using MATLAB.

### Quantification and statistical analysis

All heart rate data were analyzed using a custom-made MATLAB program. To remove the effects of the task outcome, the data when the visual feedback of result in each trial was displayed were excluded, that is, the data from the presentation of the number of consecutive successes or the total number of successes just before the presentation of the trial results were analyzed. Data from 5 trials by one participant were excluded because of the failure to acquire heart rate data.

All pupil size data were analyzed using a custom-made MATLAB program. First, we discarded 50 ms of pupil size data before and after a blink event. We then interpolated over the discarded data using a piecewise cubic Hermite interpolating polynomial (MATLAB pchip.m function). After the interpolation, the data were smoothed (smoothdata.m function). To remove the effects of gaze position during force exertion and the task outcome, the data during force exertion of participants and the data when the visual feedback of result in each trial was displayed were excluded, that is, the data from the presentation of the number of consecutive successes or the total number of successes just before their force exertion were analyzed. To control for individual differences in baseline levels and dynamic ranges, trial-wise data for pupil size were *Z* score normalized within each participant before aggregation. Data from one participant were excluded because of the failure to acquire the pupil size data.

All behavioral data were analyzed using a custom-made MATLAB program. The voltage data sampled at 1000 Hz were converted so that the cursor would be positioned at the center of the target when the participant exerted 10% of their maximum force. The total time that the cursor was completely within the target in each trial was calculated as the score. If the score was 1000 ms or more, it was judged a success, otherwise, it was judged a failure.

Following the approach of previous research,^44^ we conducted a detailed analysis of cursor movement. We divided the exertion of force in the task into three phases: an acceleration phase at the beginning of the movement, a deceleration phase when the cursor approaches the target, and a holding phase within the target. To calculate predicted errors from the acceleration phase, we first found the time of peak speed of the cursor. We then took the velocity profile from the appearance of the cursor until this time and reflected it around the time of peak speed. The resulting symmetric velocity profile was then integrated to find the displacement from the position of the appearance of the cursor, which meant a predicted endpoint based on data before the time of peak speed. The absolute value of the relative error between the predicted endpoint and the center of the target was calculated as a “predicted error from the acceleration phase”. For correction during the deceleration phase, we wanted not only a predicted endpoint but also an actual endpoint. We identified the time point at which the cursor velocity became negative following its peak, and defined the cursor position at that moment as the actual endpoint. The absolute error between the actual endpoint and the target center was defined as the actual error at the end of the deceleration phase. By subtracting this value from the predicted error from the acceleration phase, we quantified error correction during deceleration. In addition, the standard deviation of the cursor position after the cursor entered the target was calculated as a “fluctuation during the holding phase”. Reaction time for each trial was estimated based on cursor velocity data from the onset of the movement cue to the time of peak velocity. Assuming a single change point in the velocity profile, ischange.m function was used to detect the point that minimized the total cost function.^62^

Trials that met any of the following criteria were excluded from the detailed analysis of these cursor movements: “score was 0”, “force was already being exerted when the cursor appeared”, or “the speed of the cursor did not become negative”. As more than half (148 out of 263 trials) of the data from one participant in the Consecutive group met the exclusion criteria, all of that participant’s data was excluded from the detailed analysis. For all of the other participants, the number of trials that met the exclusion criteria was 42 out of 4382 trials (1.0%) in the Consecutive group and 26 out of 4367 trials (0.6%) in the Total group.

To examine how streak proximity related to physiological, behavioral, and kinematic measures (and, in the Consecutive group, the concomitant increase in psychological pressure), we transformed the trial-wise data into three within-participant streak-proximity conditions. For each participant, we identified their maximum number of consecutive successes across the experiment. Trials were then binned into: (i) Far streak proximity, comprising trials from the beginning of a run up to three successes below the individual maximum; (ii) Near streak proximity, comprising trials at two and one successes below the maximum; and (iii) Max streak proximity, comprising trials at the participant’s maximum consecutive successes. For the Total group, the maximum number of consecutive successes was truncated at nine to match the range of the Consecutive group. Within each bin, we averaged the dependent variables across trials, yielding three observations (Far, Near, Max) per participant and measure. This asymmetric binning (broader “Far” and finer bins near the maximum) was motivated by prior work showing that heart rate increases approximately exponentially as consecutive successes accumulate, such that the steepest physiological changes are expected near the end of a streak.^9^

For physiological and kinematic measures, these averaged values were submitted to mixed-design analyses of variance (ANOVAs) with a between-participant factor Group (Consecutive vs. Total) and a within-participant factor Streak proximity (Far, Near, Max). Sphericity for the within-participant factor was evaluated using the Greenhouse–Geisser epsilon. When the sphericity assumption was violated, we report Greenhouse–Geisser–corrected degrees of freedom and *p*-values. We report F statistics (with corresponding degrees of freedom), *p*-values, and generalized eta squared (η_G_^2^).

To address the continuous nature of streak progression and to test whether the main results depended on the Far/Near/Max binning procedure, we also conducted trial-level linear mixed-effects analyses using the raw number of consecutive successes as a continuous predictor. For these analyses, trials were not binned into streak-proximity conditions. For the Total group, trials with consecutive-success counts greater than nine were excluded so that the analyzed range matched that of the Consecutive group.

Specifically, we first fitted a mixed model to test whether the number of consecutive successes predominantly exhibited a monotonic (first-order) effect on the physiological metrics of interest (*y*). We then fitted two mixed models to test whether the number of consecutive successes predominantly exhibited a monotonic effect or a non-monotonic (second-order) effect on the performance metric of interest (*y*). The fixed effects were specified as:

$$\;\text{Model}\;1:y\;∼\;\beta_{0}+\beta_{1}{CS}^{1}$$

$$\;\text{Model}\;2:y\;∼\;\beta_{0}+\beta_{1}{CS}^{1}+\beta_{2}{CS}^{2}$$

with *β* as regression coefficients and *CS* as the number of consecutive successes. To draw conclusions that generalize across participants, both the intercept and the coefficients were included as by-participant random effects.^63^ We investigated whether *β*_1_ was significant in the Model1 and whether *β*_2_ was significant in the Model2. To evaluate whether the quadratic model described the score data better than the linear model, we compared the two models separately within each group using information criteria (AIC and BIC).

Perceived pressure ratings in the Consecutive group were analyzed using a one-way repeated-measures ANOVA with the within-participant factor Streak proximity (Far, Near, Max), defined in the same way as for the physiological and kinematic measures.

Planned pairwise comparisons between streak-proximity levels were conducted within each group. For consistency with the ANOVA reporting, pairwise tests are reported as F statistics with 1 numerator degree of freedom (equivalent to squaring the paired-sample t statistic), and *p*-values were adjusted using the Benjamini–Hochberg false discovery rate (FDR) procedure. For all analyses, the significance level was set at α = 0.05.

To evaluate whether the pressure-related indices provided redundant or partially dissociable information, we conducted additional exploratory analyses in the Consecutive group. For each participant, we computed change scores between streak-proximity conditions (Far to Near and Near to Max) for perceived pressure, heart rate, pupil size, and score. We first assessed pairwise correlations among changes in perceived pressure, heart rate, and pupil size. We then applied principal-component analysis to standardized change scores of the three pressure-related indices to estimate the extent to which they reflected a shared component. Finally, we tested whether single pressure-related indices, multimodal composites, or combined regression models predicted changes in score.

To characterize individual differences in late-stage performance decline, we classified Consecutive-group participants according to their Near-to-Max change in score. Participants with a negative Near-to-Max score change were classified as showing a choking-like decline, whereas participants with zero or positive change were classified as showing a clutch-like/non-decline profile. Because the Max bin was defined relative to each participant’s own maximum streak, we also conducted a conservative analysis restricted to participants who experienced at least one trial in which the current consecutive-success count was nine, corresponding to the actual goal boundary immediately before achieving ten consecutive successes. Exploratory logistic models were then used to test whether changes in perceived pressure, heart rate, pupil size, or multimodal pressure composites predicted the choking-like profile.

### Additional resources

This research is not a clinical trial; no clinical registry applies.
