# Supplementary material for: Fragile streak goals elicit multimodal pressure responses and late-stage performance decline
Source: iScience. 2026 Oct 27;29(11):118040. doi: 10.1016/j.isci.2026.118040 (PMC13735009; doi:10.1016/j.isci.2026.118040)
Supplement: Supplementary file 1 [file mmc1.pdf]

iScience, Volume *29*

Supplemental information

Fragile streak goals elicit multimodal pressure responses and late-stage performance decline

Kagari Yamada, Kazushi Tsutsui, and Kazutoshi Kudo

SUPPLEMENTAL FIGURES

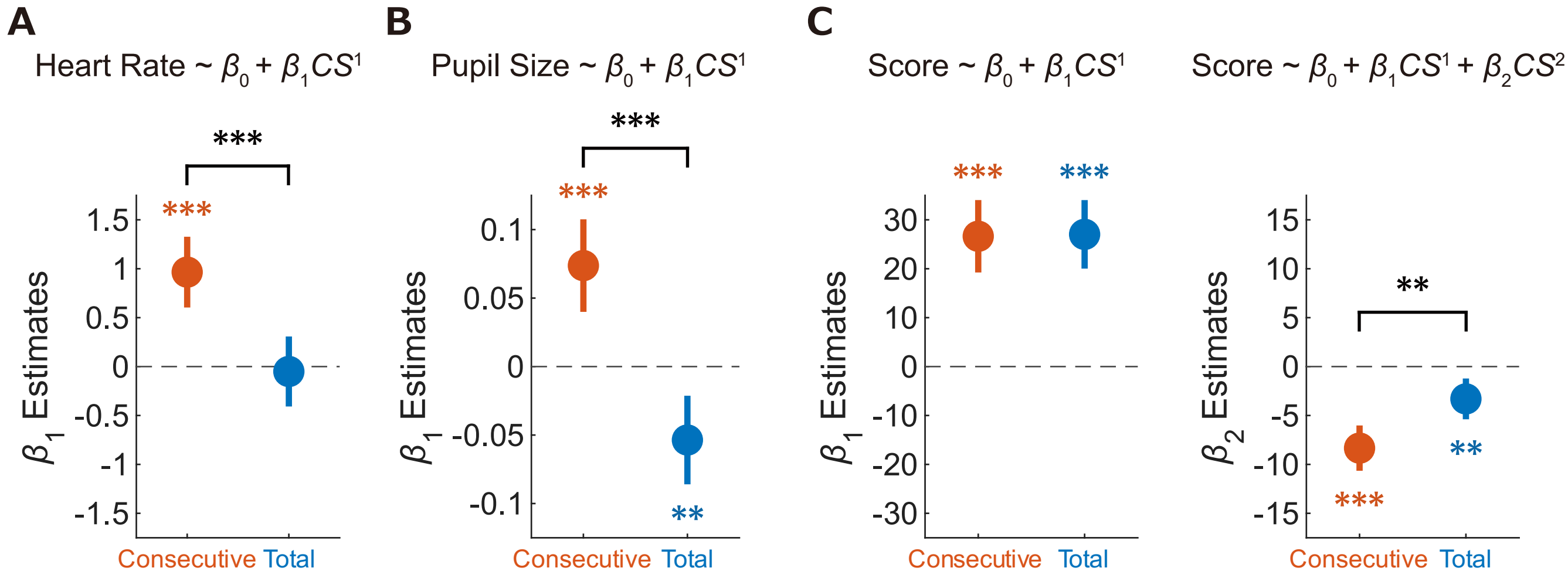

Figure S1. Trial-level mixed-effects analyses using raw consecutive-success count.

(A) Fixed-effect estimates for the linear effect of raw consecutive-success count on heart rate. Consecutive: $\beta = 0.965$, 95% confidence interval (CI) [0.604, 1.327], $p < 0.001$; Total: $\beta = -0.050$, CI [-0.408, 0.307], $p = 0.78$; Group × consecutive-success count: $\beta = -1.016$, CI [-1.524, -0.507], $p < 0.001$.
(B) Fixed-effect estimates for the linear effect of raw consecutive-success count on pupil size. Consecutive: $\beta = 0.074$, CI [0.040, 0.108], $p < 0.001$; Total: $\beta = -0.054$, CI [-0.086, -0.021], $p < 0.01$; Group × consecutive-success count: $\beta = -0.127$, CI [-0.174, -0.081], $p < 0.001$.
(C) Fixed-effect estimates for score. Left: linear coefficients from the linear mixed-effects model (Consecutive: $\beta = 26.64$, CI [19.24, 34.04], $p < 0.001$; Total: $\beta = 27.03$, CI [20.02, 34.03], $p < 0.001$). Right: quadratic coefficients from the quadratic mixed-effects model (Consecutive: $\beta = -8.34$, CI [-10.65, -6.02], $p < 0.001$; Total: $\beta = -3.30$, CI [-5.39, -1.22], $p < 0.01$; Group × quadratic term: $\beta = 5.03$, CI [1.92, 8.15], $p < 0.01$). Within-group comparisons of the quadratic and linear models: Consecutive, ΔAIC = -46.0 and ΔBIC = -33.1; Total, ΔAIC = -4.07 and ΔBIC = 8.67. Differences in information criteria were calculated as quadratic minus linear.
Points represent fixed-effect estimates and error bars show 95% confidence intervals. Red denotes the Consecutive group and blue denotes the Total group. Colored asterisks indicate whether the fixed-effect estimate within each group differed significantly from zero. Black brackets indicate significant interaction between groups for the corresponding fixed-effect estimate (**$p < 0.01$, ***$p < 0.001$).

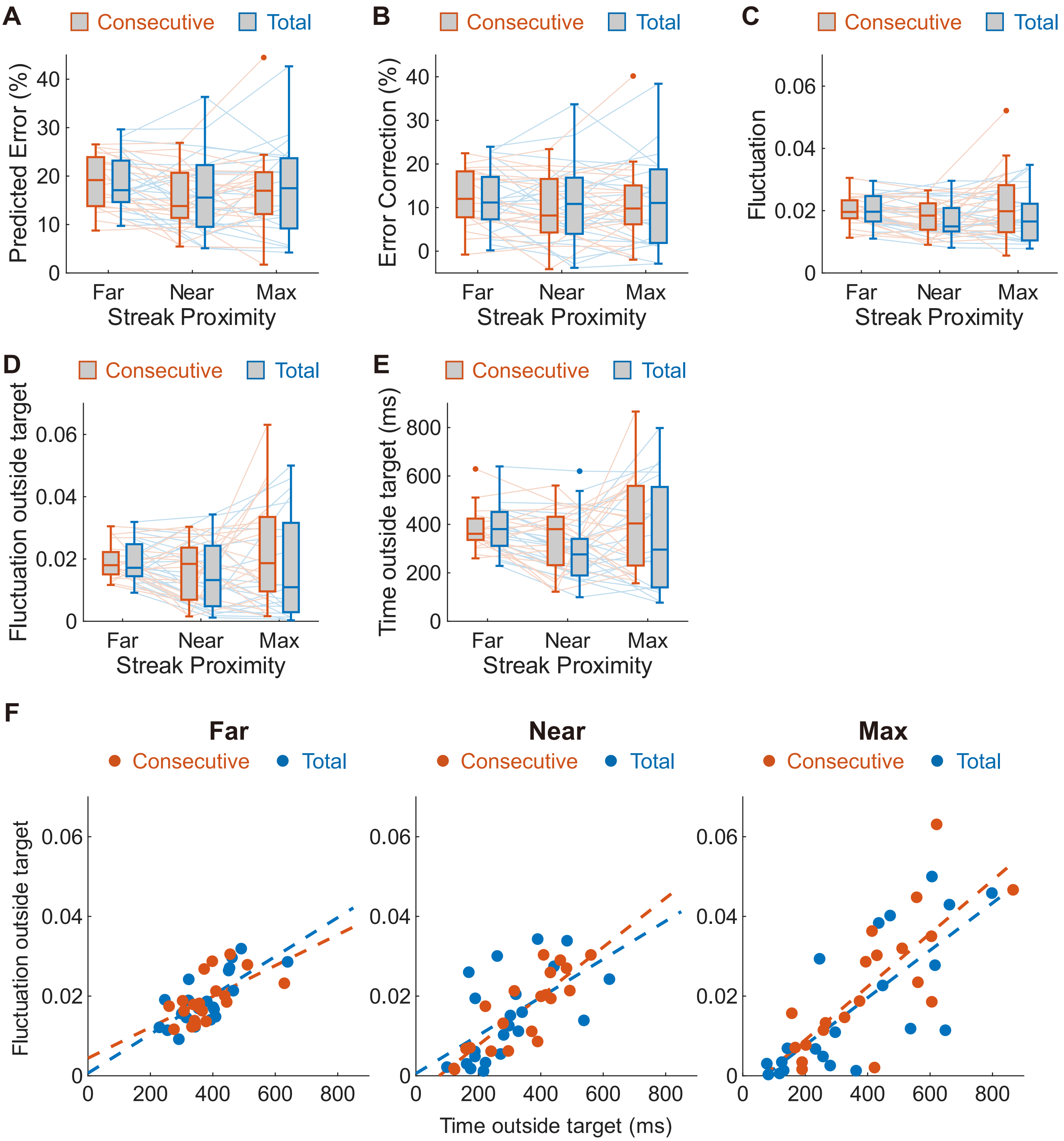

Figure S2. Indices of feedforward and feedback control as a function of streak proximity.

Panels A–E show values across the Far, Near, and Max streak-proximity conditions in the Consecutive and Total groups. Statistics are from 2 (Group) × 3 (Streak proximity) mixed ANOVAs.

(A) Predicted error from the acceleration phase. Group: F (1, 39) = 0.02, p = 0.89, η_G^2 = 0.000; Streak: F (1.84, 71.60) = 2.92, p = 0.07, η_G^2 = 0.020; Group × Streak proximity: F (1.84, 71.60) = 0.08, p = 0.91, η_G^2 = 0.001.

(B) Online error correction during the deceleration phase. Group: F (1, 39) = 0.00, p = 0.98, η_G^2 = 0.000; Streak: F (1.82, 70.90) = 0.91, p = 0.40, η_G^2 = 0.006; Group × Streak proximity: F (1.82, 70.90) = 0.16, p = 0.83, η_G^2 = 0.001.

(C) Fluctuation during the holding phase. Group: F (1, 39) = 0.28, p = 0.60, η_G^2 = 0.004; Streak: F (1.27, 49.53) = 3.10, p = 0.08, η_G^2 = 0.030; Group × Streak proximity: F (1.27, 49.53) = 0.55, p = 0.50, η_G^2 = 0.006.

(D) Fluctuation outside the target, defined as the standard deviation of cursor position during out-of-target epochs after initial target entry. Group: F (1, 37) = 0.52, p = 0.48, η_G^2 = 0.007; Streak: F (1.46, 54.16) = 2.24, p = 0.13, η_G^2 = 0.027; Group × Streak proximity: F (1.46, 54.16) = 0.47, p = 0.57, η_G^2 = 0.006.

(E) Time outside the target after initial target entry. Group: F (1, 37) = 0.77, p = 0.39, η_G^2 = 0.011; Streak: F (1.27, 46.94) = 3.45, p = 0.06, η_G^2 = 0.041; Group × Streak proximity: F (1.27, 46.94) = 0.55, p = 0.50, η_G^2 = 0.007.

(F) Correlations between time outside the target and fluctuation outside the target. Far: Consecutive, r = 0.58, p < 0.01; Total, r = 0.73, p < 0.001. Near: Consecutive, r = 0.84, p < 0.001; Total, r = 0.58, p < 0.01. Max: Consecutive, r = 0.77, p < 0.01; Total, r = 0.76, p < 0.01.

Streak proximity was defined relative to each participant's maximum number of consecutive successes (see STAR Methods). Red denotes the Consecutive group, and blue denotes the Total group. In panels A–E, boxes show the median and interquartile range; whiskers extend to 1.5× the interquartile range, and points denote outliers. Thin lines connect individual participants across conditions. In panel F, each point represents one participant, and dashed lines indicate linear fits within each group. η_G^2 denotes generalized eta squared.

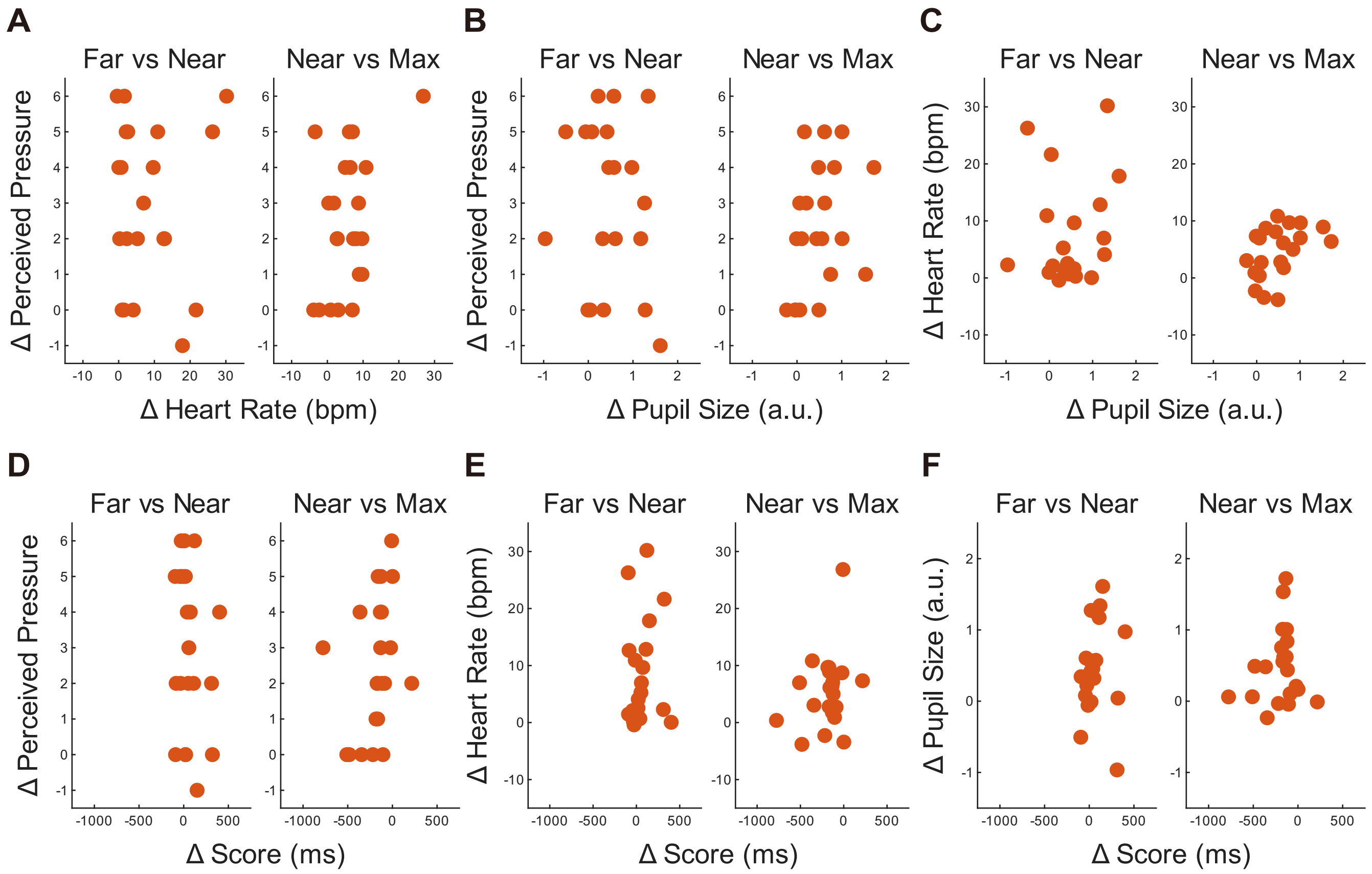

Figure S3. Pairwise associations among pressure indices and between pressure indices and performance.

Scatter plots show participant-level change scores (Δ) between streak-proximity conditions in the Consecutive group. In each panel, the left plot shows Far-to-Near changes and the right plot shows Near-to-Max changes. Each point represents one participant.
(A) Δ perceived pressure and Δ heart rate: Far to Near, $r = 0.02$, $p = 0.94$; Near to Max, $r = 0.42$, $p = 0.052$.
(B) Δ perceived pressure and Δ pupil size: Far to Near, $r = -0.15$, $p = 0.53$; Near to Max, $r = 0.36$, $p = 0.11$.
(C) Δ heart rate and Δ pupil size: Far to Near, $r = 0.15$, $p = 0.54$; Near to Max, $r = 0.43$, $p = 0.052$.
(D) Δ score and Δ perceived pressure: Far to Near, $r = -0.19$, $p = 0.42$; Near to Max, $r = 0.31$, $p = 0.17$.
(E) Δ score and Δ heart rate: Far to Near, $r = 0.10$, $p = 0.67$; Near to Max, $r = 0.29$, $p = 0.20$.
(F) Δ score and Δ pupil size: Far to Near, $r = 0.08$, $p = 0.74$; Near to Max, $r = 0.13$, $p = 0.56$.
Related analyses of these change scores (not plotted): the first principal component of standardized changes in perceived pressure, heart rate, and pupil size explained 39.8% of the variance for Far-to-Near changes and 55.3% for Near-to-Max changes. Exploratory standardized linear regression models using each single pressure-related index, all pairwise combinations, all three indices, the mean of the standardized indices, or their first principal component did not reliably predict score changes for either transition.

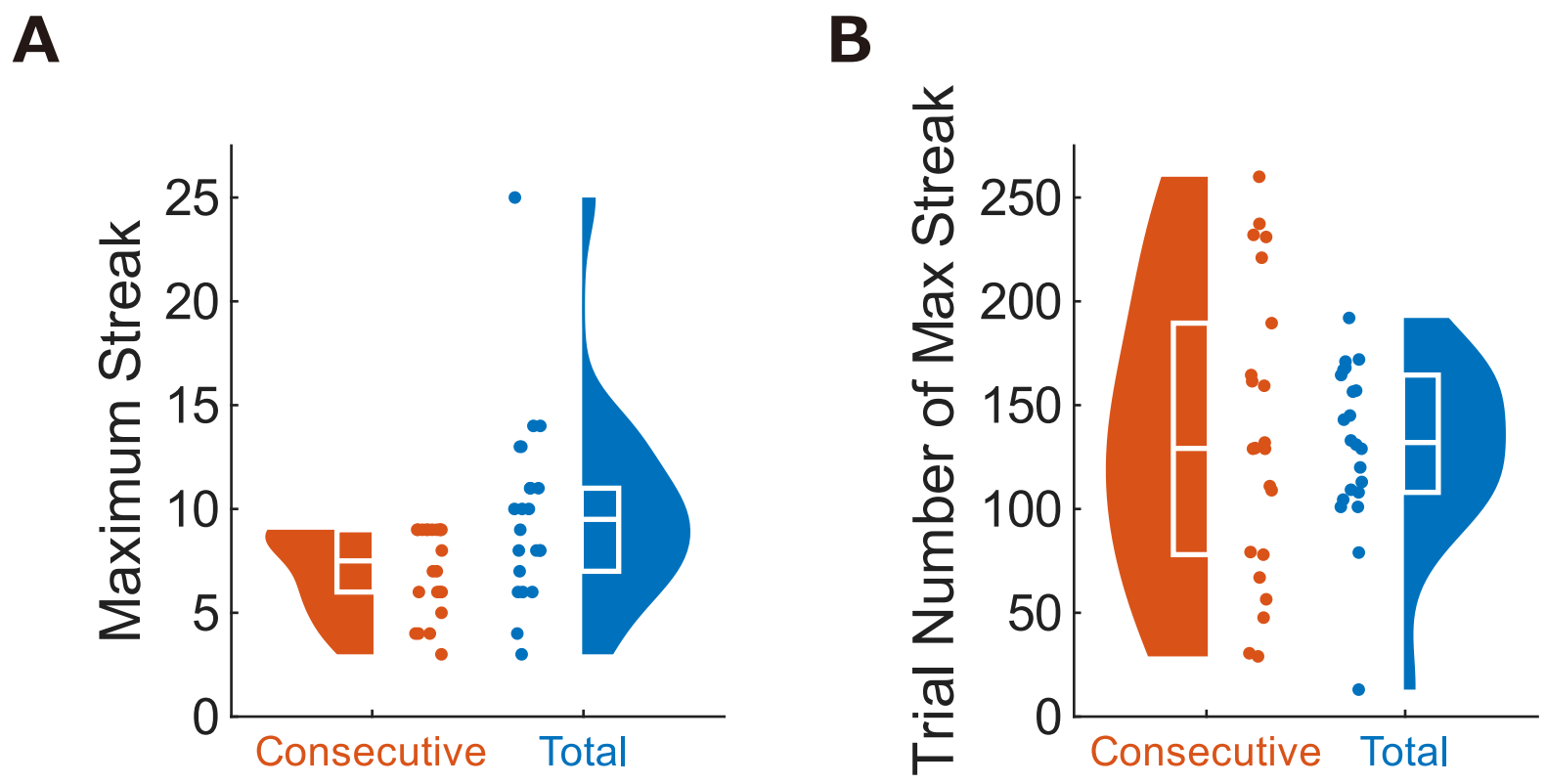

Figure S4. Maximum streak and trial timing of maximum streak.

(A) Maximum raw consecutive-success count for each participant in the Consecutive and Total groups. Consecutive: median = 7.5, interquartile range (IQR) [6.0, 9.0]; Total: median = 9.5, IQR [7.0, 11.0].
(B) Trial number at which each participant reached their maximum streak. When the maximum occurred on multiple trials, the mean of those trial numbers was used. Consecutive: 135.6 ± 69.3 trials; Total: 130.8 ± 38.8 trials (mean±SD). Between-group comparison: t (42) = 0.28, p = 0.78, d = 0.08, CI [-0.53, 0.69].
Red denotes the Consecutive group, and blue denotes the Total group. Violin plots show the distribution; box plots show the median and interquartile range; points denote individual participants.

SUPPLEMENTAL TABLES

Table S1. Number of trials at each consecutive-success count in the Consecutive group.

		Number of Consecutive Success									
		0	1	2	3	4	5	6	7	8	9	Total
Participant	1	119	40	23	13	7	5	2	0	0	0	209
	2	170	52	22	9	4	2	1	0	0	0	260
	3	10	5	4	2	2	2	1	1	1	1	29
	4	37	18	11	3	2	2	2	1	1	1	78
	5	76	30	17	10	4	3	3	3	2	2	150
	6	27	12	9	6	6	5	3	2	2	2	74
	7	116	50	32	19	10	9	7	4	3	1	251
	8	156	56	26	9	3	0	0	0	0	0	250
	9	164	66	19	3	0	0	0	0	0	0	252
	10	119	64	33	23	12	6	4	0	0	0	261
	11	130	56	34	20	6	4	2	0	0	0	252
	12	172	37	18	12	9	5	4	3	3	0	263
	13	125	51	29	17	12	9	5	4	3	2	257
	14	100	36	21	13	7	5	3	3	3	3	194
	15	95	52	33	19	10	8	1	1	1	1	221
	16	152	51	30	15	4	0	0	0	0	0	252
	17	130	66	27	13	9	4	2	1	1	1	254
	18	52	28	18	10	8	5	3	3	1	1	129
	19	159	51	26	7	3	2	2	1	0	0	251
	20	154	54	27	14	4	1	0	0	0	0	254
	21	113	58	37	21	13	4	4	2	0	0	252
	22	186	47	16	2	1	0	0	0	0	0	252
	Average	116.5	44.5	23.3	11.8	6.2	3.7	2.2	1.3	1.0	0.7	211.1

Each row represents one participant, and columns 0–9 give the number of trials at each consecutive-success count.

Table S2. Number of trials at each consecutive-success count in the Total group.

Participant	0	1	2	3	4	5	6	7	8	9	Total
	**Number of Consecutive Success**										
**1**	155	59	24	11	4	0	0	0	0	0	253
**2**	32	19	11	8	7	6	6	4	3	3	130
**3**	102	38	19	13	8	6	5	4	2	2	201
**4**	45	30	21	15	10	8	5	2	2	2	144
**5**	117	35	22	14	9	8	4	3	2	2	216
**6**	62	35	20	13	8	7	5	4	2	2	161
**7**	164	47	23	10	7	3	1	0	0	0	255
**8**	88	48	27	11	7	2	2	1	1	0	187
**9**	34	26	19	14	10	8	6	4	2	2	132
**10**	108	43	21	12	6	5	2	2	2	1	207
**11**	66	35	18	12	10	7	5	2	2	2	165
**12**	128	48	20	11	9	4	1	1	1	0	223
**13**	105	39	19	13	6	5	4	4	4	2	203
**14**	119	52	23	13	6	3	2	0	0	0	218
**15**	198	39	10	3	0	0	0	0	0	0	250
**16**	120	51	27	11	6	2	2	0	0	0	219
**17**	84	44	23	13	6	4	3	2	2	1	183
**18**	63	40	26	14	9	4	2	2	1	0	161
**19**	104	42	25	10	5	4	3	3	3	2	203
**20**	71	35	23	13	9	6	3	2	2	2	169
**21**	126	56	21	13	5	2	1	1	0	0	225
**22**	164	57	20	11	5	2	1	1	1	0	262
**Average**	102.5	41.7	21.0	11.7	6.9	4.4	2.9	1.9	1.5	1.0	198.5

Each row represents one participant, and columns 0–9 give the number of trials at each consecutive-success count.
